# Children as innovators: harnessing the creative expertise of children to address practical and psychosocial challenges of the coronavirus disease 2019 (COVID-19) pandemic – COVISION study protocol

**DOI:** 10.12688/hrbopenres.13290.2

**Published:** 2022-02-23

**Authors:** Helen McAneney, Harry Shier, Lisa Gibbs, Carmel Davies, Aoife De Brún, Kay M. Tisdall, Carmel Corrigan, Ayrton Kelly, Jacinta Owens, Onyinye Okoli, Tracey Wall, Hayda Alves, Krystyna Kongats, Revathi N. Krishna, Debbie Sheppard-LeMoine, Fernando A. Wagner, Jieh-Jiuh Wang, Carol Mutch, Thilo Kroll, Suja Somanadhan

**Affiliations:** 1 UCD School of Nursing, Midwifery & Health Systems, University College Dublin, Belfield, Dublin 4, Ireland; 2 UCD Centre for Interdisciplinary Research, Education and Innovation in Health Systems (UCD IRIS), University College Dublin, Belfield, Dublin 4, Ireland; 3 Melbourne School of Population and Global Health, University of Melbourne, Melbourne, Australia; 4 Childhood & Youth Studies Research Group, Moray House School of Education and Sport, University of Edinburgh, Edinburgh, UK; 5 Ombudsman for Children’s Office, Dublin 1, Ireland; 6 UCD Innovation Academy, University College Dublin, Belfield, Dublin 4, Ireland; 7 The George Washington University, Washington, DC, USA; 8 Children’s Health Ireland, Dublin 1, Ireland; 9 Rio das Ostras Institute of Humanities and Health, Fluminense Federal University, Rio das Ostras, Brazil; 10 Centre for Health Communities, School of Public Health, University of Alberta, Edmonton, Canada; 11 Monash University Accident Research Centre, Monash University, Clayton, Melbourne, Victoria, Australia; 12 Faculty of Nursing, University of Windsor, Windsor, Ontario, Canada; 13 School of Social Work, University of Maryland, Baltimore, Maryland, USA; 14 Ming Chaun University, Taoyuan City, Taiwan; 15 The School of Critical Studies in Education, Faculty of Education and Social work, The University of Auckland, Auckland, New Zealand

**Keywords:** Children and young people, Communities, COVID-19, Resilience, Creative arts, Pandemic, Participatory research, Psychosocial challenges

## Abstract

**Background: **We are currently in a period of transition, from the pre-COVID-19 (coronavirus disease 2019) era and the initial reactive lockdowns, to now the ongoing living with and potentially the after COVID-19 period. Each country is at its own individual stage of this transition, but many have gone through a period of feeling adrift; disconnected from normal lives, habits and routines, finding oneself betwixt and between stages, similar to that of liminality. Children and young people have been particularly affected.

**Aim: **To increase the understanding of home and community-based strategies that contribute to children and young people’s capacity to adjust to societal changes, both during and after pandemics. Moreover, to identify ways in which children’s actions contribute to the capacity of others to adjust to the changes arising from the pandemic. The potential for these activities to influence and contribute to broader social mobilisation will be examined and promoted.

**Research design: **To achieve the aim of this study, a participatory health research approach will be taken. The overarching theoretical framework of the COVISION study is that of liminality. The study design includes four work packages: two syntheses of literature (a rapid realist review and scoping review) to gain an overview of the emerging international context of evidence of psychosocial mitigations and community resilience in pandemics, and more specifically COVID-19; qualitative exploration of children and young people’s perspective of COVID-19 *via* creative outlets and reflections; and participatory learning and action through co-production.

## List of abbreviations

CBPR: Community-Based Participatory Research

CMO: Context-Mechanism-Outcomes

COREQ: Consolidated Criteria for Reporting Qualitative Research

CRAG: Children’s Research Advisory Group

e-PAR: Electronic Participatory Action Research

ESTSS: European Society for Traumatic Stress Studies

GDPR: General Data Protection Regulation

HRB: Health Research Board

JBI: Joanna Briggs Institute

MERS: Middle East Respiratory Syndrome

PCC: Population, Concept and Context

PPI: Public and Patient Involvement

PRISMA-ScR: Preferred Reporting Items for Systematic Reviews and Meta-Analyse –Scoping Reviews

PT: Programme Theory

RAMESES: Realist and Meta-narrative Evidence Syntheses: Evolving Standards

RRR: Rapid Realist Review

SARS: Severe Acute Respiratory Syndrome

SRQR: Standards for Reporting Qualitative Research

USA: United States of America

WHO: World Health Organization

Y-PAR: Youth Participatory Action Research

## Introduction

Over 100 years ago the world was dealing with the effects of the 1918 influenza pandemic, or ‘Spanish Flu’, in which an estimated 500-million people or one-third of the world’s population became infected with the H1N1 virus. Control efforts worldwide were limited to non-pharmaceutical interventions such as isolation, quarantine, good personal hygiene, use of disinfectants, and limitations of public gatherings. Nonetheless, the number of deaths was estimated to be at least 50 million worldwide ( Centers for Disease Control and Prevention, 2021). 

Pandemics are not entirely uncommon, with the movement of humans across the world, so too have infectious diseases spread. There have been several pandemics in the last 20 years, including severe acute respiratory syndrome (SARS) (2002–3), the H1N1 “Swine” flu pandemic (2009–2010), Ebola (2014–6), and Middle East respiratory syndrome (MERS) (2012-present). However, in 2020, the declaration by the World Health Organization (WHO) that the new coronavirus (SARS-CoV-2 causing coronavirus disease 2019 or COVID-19) outbreak was to be characterised as a pandemic, has been the most impactful, far reaching pandemic, in recent times ( John Hopkins Coronavirus Resource Center, 2021; World Health Organization, 2020). The world had to again revert to the need for non-pharmaceutical interventions, in the initial absence of a vaccine, to limit the spread of COVID-19. Guidance and rapidly undertaken/delivered research studies on these restrictions have occurred, for example, on self-isolation ( Arden *et al.*, 2020; Smith *et al.*, 2020), working from home ( Hadi *et al.*, 2021; Kinman *et al.*, 2020; Toniolo-Barrios & Pitt, 2021; Vyas & Butakhieo, 2021), school closures which affected over 1.5 billion (85%) of school children worldwide, liable to increase educational inequalities ( Grewenig *et al.*, 2020; Kasturkar & Gawai, 2020; UNESCO, 2021); quarantine periods with the psychological impact and perception on quality of life ( Brooks *et al.*, 2020; Lardone *et al.*, 2020), and social distancing experienced barriers and facilitators for adult ( Coroiu *et al.*, 2020) and young people ( Public Health Agency Behaviour Change Group, 2020). The restrictions (although needed) and their consequential impact, have had a profound effect on all aspects of society, including physical and mental health, and many of the direct and indirect consequences are still not fully known ( Holmes *et al.*, 2020). 

Although significant progress has been made, with at least seven different vaccines being rolled out in countries across the world as of February 2021, it will take time and resource for the global population to be fully inoculated ( World Health Organization, 2021). While efforts continue, the global population is still feeling the effects of various restriction on normal life, although to differing degrees, as countries are at differing stages of management of the pandemic. Consequently, there has been a shift from coping with the immediate effect of and restrictions placed on people due to COVID-19, to planning for the longer-term transition to living with, and the potentially considerable aftereffects of, COVID-19 ( Stark *et al.*, 2020).

It has been shown that children and young people have been particularly affected by the restrictions of isolation, prolonged confinement and uncertainty placed on them by the COVID-19 pandemic, all of which is set against the backdrop of increased prevalence of mental health issues in certain groups ( Brooks *et al.*, 2020; Crawford, 2021; Dalton *et al.*, 2020; Ford *et al.*, 2020; Green, 2020; Larcher *et al.*, 2020; Scottish Government: social research, 2020; Wang *et al.*, 2020). Adolescence (the stage between 10 years and adulthood) is a period of life when social connections and peer interactions are particularly needed, and although the use of digital technologies may mitigate some of the negative effects of social distancing, it does not fully replace face-to-face social contact ( Orben *et al.*, 2020). Studies have reported increased educational inequalities caused by school closures ( Grewenig *et al.*, 2020), the effects of social deprivation on adolescent development and mental health due to the removal of regular social connection ( Orben *et al.*, 2020), and the added strain placed on family units ( Scottish Government: social research, 2020). However there have also been noted positivity from the restrictions, such as added time together with family members, especially for children and parents learning together, increased creativity, displays of empathy and generosity due to the pandemic, and social integration, with families and communities engaging in a high level of social cohesion ( Hupp, 2020; Karunathilake, 2020; Kim, 2020; Nelson, 2020).

Over recent years there has been a surge of international interest in resilience science in children and young people, in consequence to natural disasters, political violence, but also pandemics ( Masten, 2014; Masten & Barnes, 2018). Resilience theory is a conceptual framework for understanding how some individuals can bounce back after experiencing an adverse situation. One marked way that children and young people have overcome ‘cabin fever’ has been through the creative process and channelling of emotion through other/alternative mediums ( Crawford, 2021). This is nothing new, as creative art therapies, an umbrella term for healthcare professions that use the creative and expressive process of art making to improve and enhance the psychological and social well-being of individuals of all ages and health conditions, has been implemented in previous life changing experiences, as well as being adapted now to the COVID-19 pandemic ( Carswell *et al.*, 2021; Dieterich-Hartwell & Koch, 2017; Miller & McDonald, 2020; Shafir *et al.*, 2020). This too has been seen in the research area of post-disaster recovery, where mental health risks can be mitigated, at the community level, through protective factors such as social capital- the direct and indirect result of social connections or social networks, along with cooperation to achieve a better social or economic outcome ( Aldrich, 2012; Putnam *et al.*, 1993), and with connections with community groups ( Gallagher *et al.*, 2019). Not only does the creative voice allow personal evolution and healing to occur, but it can aid social mobilisation, the process of bringing together the societal and personal influences to raise awareness and need for health care, and cultivate sustainable individual and community involvement ( World Health Organization, n.d.). There are examples of children contributing to local efforts to promote hope, community efficacy and connectedness, through chalk messages on pavements and drawings and messages in windows ( Murray-Atfield, 2020). This is consistent with previous examples of children’s contribution to household and community level recovery from major emergencies ( Freeman *et al.*, 2015; Peek, 2008) and is an essential part of any disaster risk mitigation strategy ( Hoffmann & Blecha, 2020).

### Overall study aims and objectives

The aim of this project is to increase the understanding of home and community-based strategies that contribute to children’s capacity to adjust to societal changes, both during and after pandemics (particularly strategies addressing their sense of safety, calm, hope, self and community efficacy, connectedness). Moreover, the project aims to identify ways in which children’s actions contribute to the capacity of others to adjust to the changes arising from the pandemic. The potential for these activities to influence and contribute to broader social mobilisation will be examined and promoted.

This project will be realised through five distinct objectives aligned to four work packages:

1. To have oversight and the voice of key stakeholders (children and young people) embedded within all aspects of the project through Public and Patient Involvement (PPI). This will be through experience-based co-design such that only relevant and useful outputs will be delivered, that is, *via* co-production, co-design and co-create.
2. Undertake a rapid realist review (RRR) of international literature and practice to identify, appraise and understand how protective mechanisms mitigate the psychosocial risks children face in a pandemic.
3. Carry out a scoping review of international literature to establish children and young people’s contributions to building community resilience during the COVID-19 pandemic.
4. Investigate children and young people’s perspective through the collection of their reflections on creative outlets and processes as a result of and related to COVID-19 experiences.
5. Based on children's experiences during COVID-19, their needs and priorities, accessible communications (e.g., policy briefings, video or still animation, comic strips) will be co-produced to convey key learnings and messages agreed as key through the co-production process. These could be for use in policy, practise, and the community for future reference or in similar pandemic situations.

## Protocol

### Study design

**Figure 1.**
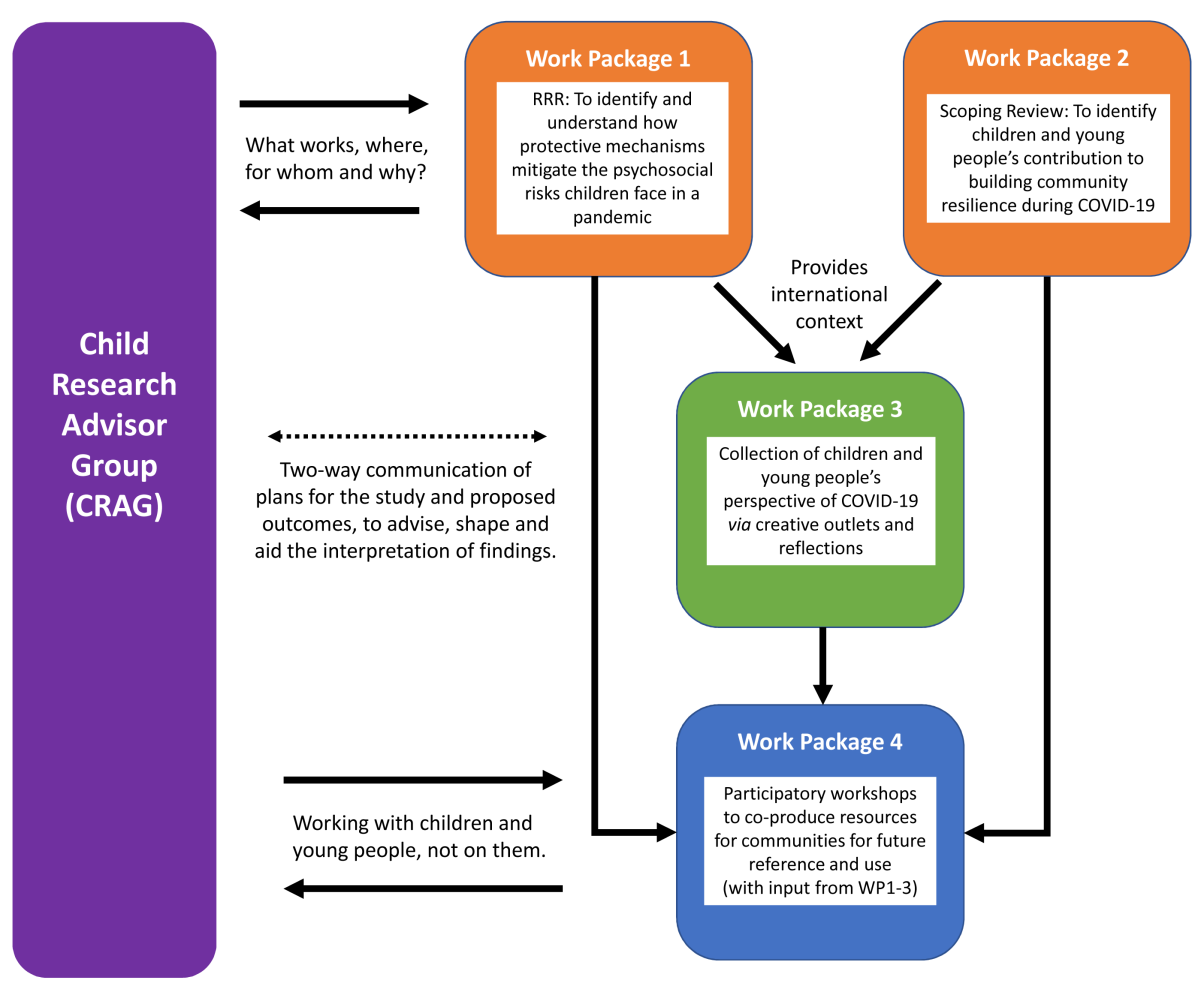
Overview of the research process of the COVISION study. RRR=rapid realist review; COVID-19=coronavirus disease 2019.

The study design is mapped out over four interlinked work packages, including two syntheses of literature (a RRR and scoping review) to gain an overview of the emerging international context of evidence of psychosocial mitigations and community resilience in pandemics, and more specifically COVID-19, qualitative exploration and participatory learning and action through co-production. Figure 1 illustrates the key elements of the work packages and the workflow of the study design. 

The overarching theoretical framework of the COVISION study is that of liminality. Although the concept of liminality has its origins in “rites de passage” (or “rites of passage”) and contributed to the development of transition theory ( Van Gennep, 1960), more recent usage has broadened to describe political and cultural change, including situations applied to entire societies going through a crisis ( Thomassen, 2009 p19), as well as individual health and illness situations ( Somanadhan & Larkin, 2020). We are currently in a period of transition, from the pre-COVID-19 era and the initial reactive lockdowns, to now the ongoing living with and potentially the after COVID-19 period. Each country is at its own individual stage of this transition, but many have gone through a period of feeling ambiguous and in a sense of no man’s land, finding oneself betwixt and between stages, similar to that of liminality ( Turner, 1975). Cook-Sather describe liminality as the stage in the rite of passage defined as, “the place within which that transition takes place, and the state of being experienced by the person making the transition” ( Cook-Sather, 2006, pp. 110). The rites of passage theoretical framework will help to understand how children and young people adjust to societal changes, both during and after pandemics, and have a role in the wider capacity of communities to adjust and thus social mobilisation.

**Figure 2.**
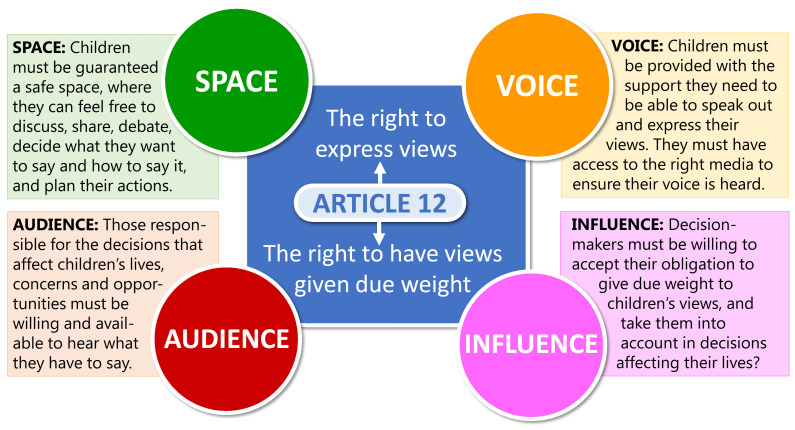
The Lundy model of participation, Lundy (2007). The four key elements of *space*, *voice*, *audience*, and *influence* are essential for children’s participation in decision-making to be effective, meaningful, and compliant with their rights.

To achieve the aim of this study, a participatory health research approach will be taken. That is, both researchers and participants work in co-operation and “research is not done “on” people as passive subjects providing “data,” but “with” them to provide relevant information for improving their lives” ( ICPHR) and it is viewed as a partnership between stakeholders and reflexively will be drawing upon their experience ( Green & Thorogood, 2009). As a pragmatic approach the Lundy (2007) model of participation will be following were the four key elements of *space*, *voice*, *audience*, and *influence* are essential for children’s participation in decision-making to be effective, meaningful, and compliant with their rights (see Figure 2). The analytical tool of Shier (2019) will then aid the study to develop partnerships with children (summarised in Table 1). The tool can be used both to plan a process for engagement with children in research, or to monitor and evaluate a project during or after implementation.

**Table 1.** Analytical tool to help researchers develop partnerships with children (from Shier, 2019).

		Dimension of decision-making power or control of children				Who is involved and/ or excluded?
		Not involved	Children are consulted	Collaboration with researchers	Children direct and decide	
**Phases of the research process**	Deciding on the research question		Children asked about problems that concern them.	Children and adults jointly define research question.	Children choose their own research question.	Who has a say in the research question?
	Designing the research and choosing methods		Children consulted on what research methodology to use.	Children and adults deliberate and jointly decide on the methodology to use.	Children decide what methodology they want to use.	Who is invited to get involved in the research design?
	Preparing research instruments		Children consulted on (and perhaps test) research instruments before use.	Children and adults work together on design of research instruments.	Children create their own research instruments.	Who gets to work on the research instruments?
	Identifying and recruiting participants		Children asked to advise on recruiting participants.	Children and adults jointly identify and recruit participants.	Children identify and recruit research participants.	Who has a say in choosing participants?
	Collecting data		Research involves adults interviewing children or surveying their opinions.	Children and adults collaborate on data-gathering activity.	Children organise and carry out data collection activities.	Who gets involved in data collection?
	Analysing the data and drawing conclusions		Adults show preliminary findings to children and ask for feedback.	Children and adults work together to analyse data and determine conclusions.	Children analyse data and draw their own conclusions.	Who has a say in what the conclusions are?
	Producing a report		Adults consult children on aspects of the final report.	Children and adults work together to produce a report.	Children produce their own report in their own words.	Who gets credit for the report?
	Dissemination of the report and its findings		Adults consult children on how to disseminate findings.	Children and adults collaborate on dissemination and awareness- raising activities.	Children undertake activities to disseminate their findings.	Who is actively involved in dissemination?
	Advocacy and mobilisation to achieve policy impact		Adults consult children about possible advocacy actions.	Children and adults work together on plans for advocacy and mobilisation.	Children develop and implement an action plan for advocacy and mobilisation.	Who is active in follow-up campaigning and advocacy?

*The core matrix of the analytical tool by Shier (2019). Rows depict phases of the research process and columns the decision-making power or control that children may have. The last column provides prompts to discern who is included and/or excluded from the process. This table has been reproduced with permission from Shier (2019).*

All study materials including consent forms and assessment tools can be found as extended data ( McAneney, 2021).

### Ethical approval

This research has been approved by the Human Research Ethics Committee, University College Dublin [LS-21-55-Somanadhan].

### Children’s Research Advisory Group (CRAG)

**Objective**

To frame a collaborative process involving work in partnership with children ^1^. 

**Method**

To achieve the objective a Children’s Research Advisory Group (CRAG) will be set up to guide the implementation of the project. The CRAG will be formed of children and young people aged 10–17 years old, to advise the researchers on the formulation of research questions, appropriateness of methods, design of data-gathering instruments, analysis and interpretation of findings, and/or design of dissemination materials and methods. Members ( *n*=10–15 children and young people) of an advisory group act in an expert role to add value to the research, and in general do not provide data for the research. Advisory groups allow the key stakeholders to be engaged in every stage of the research, as appropriate to the circumstances, and with a considerable amount of flexibility.

Following the Lundy model of participation (2007), which is illustrated in Figure 2, the CRAG will provide a *space* where children can develop and express their *voice*, with the adult project team being their first *audience* and thus enabling them to *influence* the implementation of the project ( Lundy & McEvoy, 2012; Moore *et al.*, 2016). The CRAG will have direct input into the rapid realist review (WP1) and the participatory workshops to co-design deliverables (WP4). The latter provides a further *space* allowing children’s *voice* to reach a wider *audience* and *influence* the outcomes and longer-term impact of the project. 

Given the global reach of this study, four advisory groups are proposed: one comprising children and young people from Ireland and Scotland, one from Australia and New Zealand, one from the USA and Canada, and another from India. These groups will meet on a regular basis (proposed as monthly) throughout the study. The CRAGs will progress through the scheme of work proposed by the researchers in relation to the work-packages of the study, such that forthcoming tasks are known, and the CRAG members can be prepared in advance, with training, knowledge, and support as and were required. Consequently, the input and feedback from CRAG will be informative, meaningful, and worthwhile.

In addition to the Lundy model, the research team will also draw on Shier’s analytical tool ( Shier, 2019; summarised in Table 1), which will aid the development of partnerships with children and adolescents. Given the implementation of this project under pandemic conditions, the CRAG will meet virtually rather than physically, using appropriate and accessible technology to facilitate this ( Cuevas-Parra, 2020).

**Expected outcome**

The on-going involvement of the CRAG will inform and shape the research direction and outputs, making it a stronger embodiment of work.

### Work package 1: Identifying and understanding how protective mechanisms mitigate the psychosocial risks children face in a pandemic

**Objective**

To identify and understand how protective mechanisms mitigate the psychosocial risks children face in a pandemic, a Rapid Realist Review (RRR) will be conducted. Our focus is to identify the range of interventions that positively affect children's social support and resilience. While the review expands the parameters to include literature on any pandemic, the results will be applied within the current COVID-19 pandemic's narrower context.

The purpose of this review is to develop an initial programme theory (PT) that will:

- Identify what social interventions have been demonstrated to mitigate the psychosocial risks children face in a pandemic.
- Synthesise explanations of how context and mechanisms enable social interventions to achieve positive outcomes.
- Describe the contextual issues that influence how these interventions work.

The findings will directly inform the following work packages of the project that involves an empirical exploration (WP3) followed by co-production and co-creation of resources (WP4). The programme theory will also be represented in a publication to inform broader international debates in this field.

**Method**

The rationale for a realist approach for this aspect of the study is built on widespread evidence showing that conventional systematic reviews to determine whether interventions work (or not) often result in limited answers ( Pawson *et al.*, 2005). Systematic reviews control contexts, whereas realist approaches embrace contextual complexity. In this way, a realist approach seeks to provide more in-depth explanations of why and how interventions work ( Pawson *et al.*, 2005; Pawson & Tilley, 1997; Pawson, 2006). This enquiry area is critically important within broader policy and research debates on the challenges associated with the failure of interventions to achieve or sustain the positive outcomes initially intended ( Damschroder *et al.*, 2009; May *et al.*, 2016).

In line with the work of Pawson on realist evaluation ( Pawson, 2002), a RRR develops programme theory to better understand how complex interventions work. It unpacks the dynamic complexities and makes explanatory connections about how context and mechanisms influence outcomes. The programme theory is presented using the convention of Context-Mechanism-Outcomes (CMOs) configurations ( Pawson & Tilley, 1997; Saul *et al.*, 2013). It is useful for knowledge users and policymakers as it helps to illustrate 'what works, for whom, in what contexts, to what extent, and most importantly, how and why?' It thus gives policymakers greater foresight on how well a particular intervention is likely to work when implemented ( Davies *et al.*, 2019; May *et al.*, 2016; Pawson *et al.*, 2005; Shé *et al.*, 2018) and supports learning from what does not work and why.

A RRR is particularly appropriate for this project, as it seeks to address the emergent effects of COVID-19 on children's wellbeing, and requires a rapid, evidence-based policy response ( Saul *et al.*, 2013).

**Figure 3.**
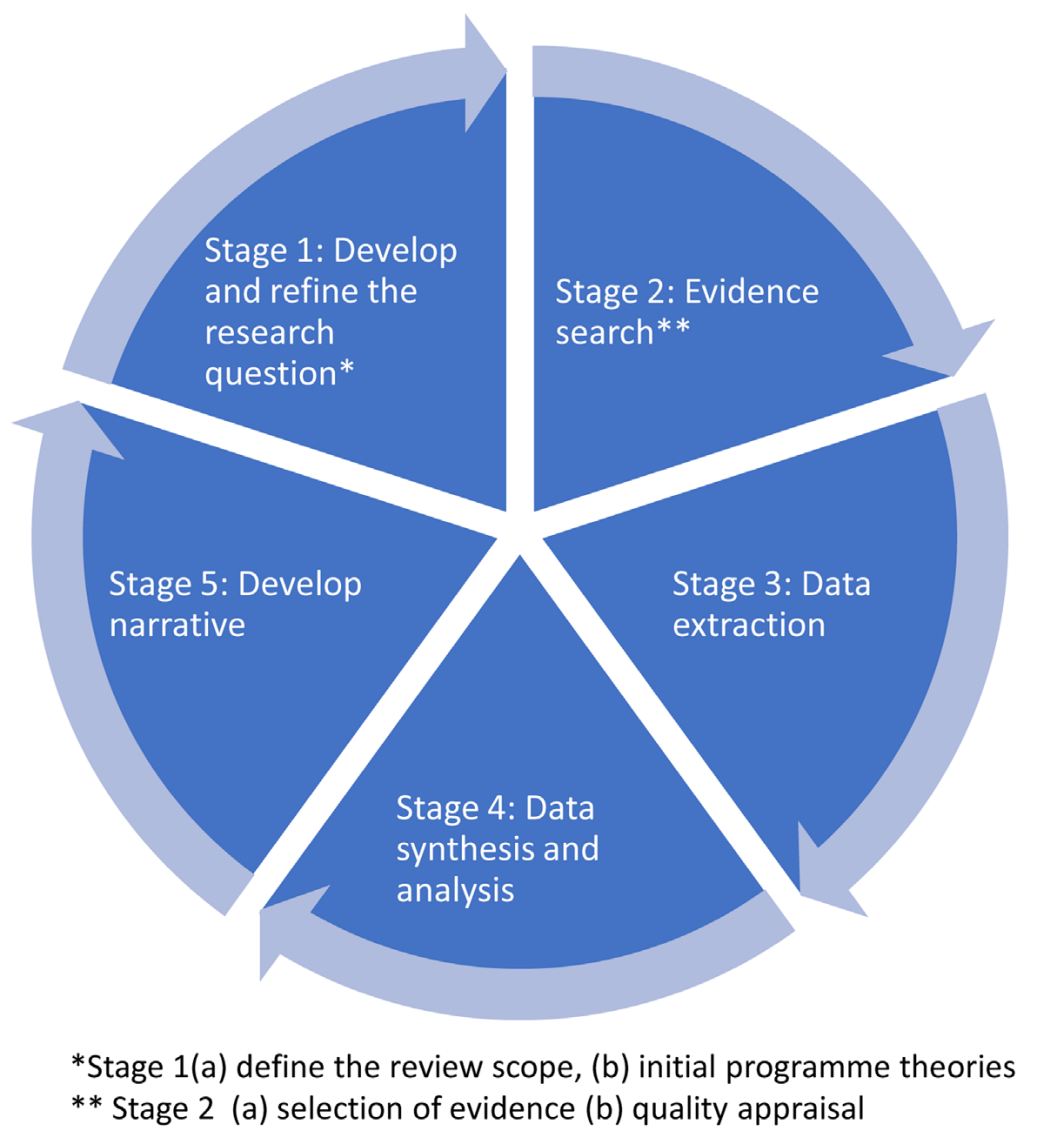
Core stages of the rapid realist review process, which is iterative and non-linear in nature.

The ‘Realist and Meta-Review Evidence Synthesis: Evolving Standards’ (RAMESES) II will be followed for this review ( Wong *et al.*, 2016). This requires a number of iterative steps, including developing and refining a research question, quality appraisal, data extraction, and evidence synthesis, as illustrated in Figure 3. The review will adopt broad criteria for inclusion of sources of evidence, both quantitative and qualitative for programme theory development.

To achieve this, a content expert panel will be established, with representation from researchers and knowledge users from policy, research, education, health, and advocacy groups. CRAG members will be invited to participate in this panel to provide their expert opinion. The role of the expert panel will be to (i) clarify and agree on the study scope, (ii) the research question, (iii) literature search strategy, (iv) inclusion/exclusion criteria and (v) refining and agreeing the programme theory. The expert panel involvement thus strengthens the overall quality and relevance of the review, ensuring that the research question is relevant and in line with social and policy priorities. Availing of the experience of policymakers and knowledge users ensures the results are outcomes-focused, taking account of evidence synthesis (research literature) and expert panel discussion (local contextual knowledge) ( Davies *et al.*, 2019; Saul *et al.*, 2013; Shé *et al.*, 2018), which enhances the relevance and practical application of the results ( Pawson, 2002).

**Expected outcome**

The results of the synthesis will be written up according to the RAMESES II standard for reporting realist reviews and published in a peer reviewed journal. Further, a plain English summary and stakeholder friendly summaries (e.g., infographic) will be co-produced with the CRAG. The results from the synthesis will provide an international context for WP3 and 4. 

### Work package 2: Identifying children and young people’s contributions to building community resilience during the COVID-19 pandemic

**Objective**

To identify and establish children and young people’s contributions to building community resilience during the COVID-19 pandemic.

**Method**

Scoping reviews are a valuable approach for evidence synthesis ( Munn *et al.*, 2018), especially when evidence is emerging or to explore the nature and diversity of the evidence/knowledge available ( Armstrong *et al.*, 2011; Aromataris & Munn, 2020). For this work package, the methodology of a scoping review for evidence synthesis has been chosen due to the ongoing and rapidly emerging evidence related to COVID-19, as a more systematic approach would not yet be appropriate. The scoping review will collate both published peer reviewed literature and unpublished grey literature on children and young people’s contributions to building community resilience during the COVID-19 pandemic.

The methodological framework for this review will be based on that of Arksey & O’Malley (2005), which was built on and refined by Levac *et al.* (2010), and the Joanna Briggs Institute’s (JBI) methodology for scoping reviews ( Peters *et al.*, 2020). The review will follow the standard six stages: (1) identifying the research question; (2) identifying relevant studies; (3) selecting studies; (4) charting the data; (5) collating, summarising, and reporting the results and (6) consulting with relevant stakeholders ( Levac *et al.*, 2010). Rigour in reporting will be ensured by using Preferred Reporting Items for Systematic reviews and Meta-Analyses extension for Scoping Reviews (PRISMA-ScR) Checklist ( Tricco *et al.*, 2018).

***(1) Identifying the research question***

This process begins with establishing a review team consisting of individuals with experience in the topic and research synthesis ( Levac *et al.*, 2010), but given the emergent situation of COVID-19, experience in disaster recovery and community resilience will be used as a proxy. The scoping review question guides and directs the development of the review process. In this review, we will utilise the population, concept and context (PCC) framework ( Peters *et al.*, 2020). The preliminary review question is: What evidence-based resilience interventions (C) are used for and by children and young people (P) during and after a pandemic in community settings (C). A secondary objective will be to focus on what lessons can be learnt from children and young people’s contributions focusing on their rights, participation, and resilience during a pandemic.

***(2) Identifying relevant studies***

A systematic search of electronic databases, limited by year from 01 January 1990 to present, will retrieve relevant published, primary research literature. Databases to be searched will include: CINAHL, EMBASE, MEDLINE, PsycINFO, Web of Science, and the Cochrane COVID study register. Grey literature will be collated from searching the GreyLit and OpenGrey databases, as well as reports known to the authors. Further, forward and backward citation searches will be conducted to fully scope out the literature. Since it is crucial to have a global representation, no language exclusion will be applied in the initial search. Searching all languages will allow the numbers of potentially eligible non-English publications to be identified, and to determine whether a source of language bias might have otherwise resulted. Where possible, English translation of the abstract and/or paper will be sourced, or expertise from the multi-national authorship will be utilised for translation.

The preliminary search for research studies will be focused on the current COVID-19 pandemic, and then widened to include other of the largest known pandemics caused by an infectious disease. For example, “Dengue Fever”, “The Kivu Ebola epidemic”, “Zika virus epidemic”, “Swine Flu epidemic”, SARS and MERS. A systematic search of the peer-reviewed literature using medical subject headings (MeSH) terms, keywords, and database categorization will be conducted (see Table 2). This iterative process will involve broad search terms, including combinations, truncations, and synonyms of “Pandemic” as well as more specific searches focused on “COVID-19,” “Coronavirus,” “SARS-CoV-2”, “Coronavirus disease”. The other terms to focus the searches will include terms such as “Child”, “Children”, “Young people”, “Young adult”, “adolescents,” “youth,” “teenagers,” “Creativity,” “arts,” “participation,” “youth engagement,” “youth involvement,” “participatory research” (will cover Y-PAR, CBPR, PAR, e-PAR), “action research” and “participatory practices.” The searches will look for the features provided in Table 2, anywhere in the text, but will be limited to primary studies published after 1990. Within each of the search term features, the keywords are combined using the ‘OR’ Boolean operator between them, allowing for a broader search to be performed. The ‘AND’ Boolean operator between the features then narrows the search as one term from each feature is specifically required.

**Table 2.** Keyword search terms for the scoping review of work package 2.

Feature	Search Terms
(P) Population	Child* OR Young people OR Young person OR Adolescen* OR Teenage* OR Youth
(C) Context	Pandemic OR COVID* OR SARS-CoV-2 OR Coronavirus
(C) Concept 1 Concept 2	Resilience OR Resiliency OR Resilient Intervention OR Co-design OR Co-production OR Participatory OR Participative OR Strategy OR Strategies

***(3) Selecting studies***

Search results will be imported into the Covidence software and duplicates will be removed. The screening process will be completed by two independent persons (HS, SS), with any conflict decided by a third reviewer (HMcA). Firstly, titles and abstracts will be screened, after which full articles will be read and screened. Relevance for inclusion will be based on the age of participants (10–17 years old required), availability of full text, and of any study design but must be primary research, i.e., not review articles.

***(4) Data collection***

The second stage of screening results will be carried out independently by two researchers using a data charting form ( Levac *et al.*, 2010; Peters *et al.*, 2020). A sample chart for collecting the data, which is adapted from the Cochrane collection template and informed by Nicholson *et al.* (2019), will be utilised as a part of the scoping review. Examples of data to be noted or extracted will include country, number of participants, qualitative methodology and methodological rigour. Any conflicts or differences will be discussed by the two reviewers, and when disagreement still results, a third reviewer will adjudicate.

***(5) Data summary and synthesis of results***

The data will be collated and summarized in accordance with the overall aim and objectives of the scoping review. Results of the study screening process will be presented in a PRISMA-ScR flow diagram ( Tricco *et al.*, 2018). Charted data will be synthesised quantitatively and qualitatively, and potentially using a narrative synthesis ( Barnett-Page & Thomas, 2009). Review results will conclude with an overview of research limitations, knowledge gaps and areas that have been under-researched to inform directions for future research and considerations for policy and practice. The processes for collecting and analysing data and the reasoning behind all data handling and analysis decisions will be detailed in the final manuscript.

***(6) Consultation***

Expert consultation is an integral part of the scoping review process because it allows key stakeholders with expertise in research, policy, and practice to participate in the review, which improves methodological rigour and applicability ( Levac *et al.*, 2010). Consultations will occur with national and international experts on children and young people’s participation, disaster recovery, children’s rights, and children's health, as well as with the CRAG members who will be informed of the findings and offered the opportunity to input into the articulation of the scoping review. This consultation with key stakeholders, experts, and key informants will be undertaken to clarify potential gaps in the review or ongoing related studies or interventions that are not included in the review.

**Expected outcome**

The results of the synthesis will be written up according to the PRISMA-ScR standard for scoping reviews and published in a peer reviewed journal. Further, a plain English summary and stakeholder friendly summaries (e.g., infographic) will be co-produced with the CRAG. The results from the synthesis will provide an international context to inform the work conducted in WP3 and 4. 

### Work Package 3: Data collection of children and young people’s perspective of COVID-19 *via* creative outlets and reflections

**Objective**

To investigate children and young people’s perspective through the collection of their reflections on creative outlets and processes as a result of and related to COVID-19 experiences.

**Method**

***Context and setting***. Children and young people aged 10–17 years of age from anywhere in the world are eligible to participate in this study. Informed consent will be sought from parents (or guardians) as well as assent from the children themselves. The survey/data submission will be hosted online *via* Qualtrics, which is GDPR (general data protection regulation) compliant.

**Figure 4.**
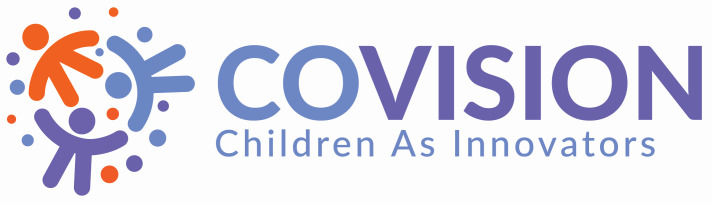
Study logo designed in partnership with children, to represent global reach, partnership, sharing and solidarity.

***Sample and recruitment***. The data collection for this work package of the study will be advertised through social media, and further disseminated across establish globally connected networks of the project team and key stakeholders. This will include a flyer with information of the study details and how to get involved, using our study logo to promote our study (see Figure 4). Further, a video to advertise the COVISION study will be developed and shared over social media, as well as on the dedicated study website ( www.covision.ie). As the study will be promoted and performed online, the sample will be purposive, rather than representative. We envisage a sample of *n*=20–50 responses, as is typical for studies promoted in this way. However, should we have over 150 responses or reach data saturation, we will close the data collection early. We further acknowledge the limitations of using a digital medium to collect the data, given the digital disparity that exists across the world.

***Data collection***. The primary outcome will be collection of creative works by children and young people in response to their own experience of the impact of, or related to, COVID-19. In essence it will be the collation of how children have been experiencing the COVID-19 pandemic over the last 12 months. In addition to the creative outlet (whether the modality is photography, drawing, writing (including song writing and poetry) or painting), the child/creator will be asked to provide a verbal or written narrative to talk through their submission. That is, to talk about what they have drawn, painted or photographed, to probe into what does it illustrate, or tell us more about the content and your thoughts when you created this. The submission may be new creative pieces, recent or even older, but must be as a response to the COVID-19 pandemic. If a narrative of the creative piece is given visual/audio-visually, this will be transferred verbatim to a transcript.

Secondary outcomes to be collected will include: (i) gender (ii) age (iii) ethnicity, (iv) country, which will provide contextualisation of the data and allow for potential comparative case studies across countries. An important factor to consider is the level of restrictions experienced by the child at the time of their entry, and this will be determined according to the following scale: None (no change to normal living routine); Minimal (e.g., still attending school, but not allowed to play with friends after school); Some (e.g., still attending school, but not allowed to play with friends after school and needed to stay 1.5 –2 meters away); Moderate (e.g., home schooling, able to go out to parks); Severe (not allowed to leave the family home); Other. Lastly, the person to whom instigated the creative process will be sought to determine if it was a) self-inspired b) teacher c) parent/carer or d) other e.g., resulting from a competition. 

Further details are given in the extended data, where a blank survey has been provided, as well as the information sheets and consent and assent forms. We would envisage the data collection to be open for 4–6 months, although this may be extended or reduced depending on response rates.

***Data analysis***. The data analysis will focus on the narrative description of the creative piece, which will be transcribed (if required) and thematical analysed. The software NVivo will be utilised to assist the team in analysis. The rapid realist and scoping review findings will provide an initial framework to be applied to the narrative descriptions/reflections. Data will be coded in line with this framework, and sub-codes further developed as required. To aid in the coding process, a familiarisation process with the collected data will occur, in which two independent analysts will read transcripts, with refinement of thematic coding framework if needed. In addition, the CRAG will be consulted during the development of the coding frame and thematic analysis, with discussions and input to ensure relevance of and accuracy of interpretation of data.

As the sample size may not be large (possibly only 30–40 pieces), it may not be possible to obtain the usual qualitative methodological rigour of data saturation or triangulation ( Green & Thorogood, 2009). However, every effort will be made to obtain a reasonable and varied sample.

As is usual with the collection of data, full data analyse will only occur after data collection has been terminated. However, the number of submission and periodical cursory look at data will be warranted to determine if data collection should terminate early. Descriptive information will be summarised in tables, with frequency/percentages and or mean/standard deviations. Subsequent sub-analysis of themes by country, age, gender or restriction levels will be investigated if possible.

As this work package is qualitative in nature, the standards for reporting qualitative research (SRQR) will be adhered to ensure rigour ( O’Brien *et al.*, 2014).

**Expected outcome(s)**

A report of the findings will be presented to the CRAG, and an infographic and short summary disseminated through the study website. These summaries and findings will provide the initial starting point for WP4. There is the potential to also provide an exhibition of creative pieces, *via* the study website, dependent on additional individual consent. Peer reviewed publication(s) of the study findings will also be developed to disseminate findings internationally.

### Work Package 4: Co-design of outputs through participatory workshops

**Objective**

Based on children's experiences during COVID-19, their needs and priorities, accessible communications (e.g., policy briefings, video or still animation, comic strips) will be co-produced to convey key learnings and messages agreed as key through the co-production process. These could be for use in policy, practice, and the community for future reference or in similar pandemic situations.

**Method**

As articulated by Goodyear-Smith *et al.* (2015), the implementation of participatory and co-designed produces has the defining feature of being emergent and adaptive, making pre-specification difficult to impossible. Nonetheless, Goodyear-Smith *et al.* (2015) provide guiding principles to aid in the process of participatory research which will be followed; for instance, the setting and establishment of ground rules for co-design and communicated to all members, both the study team and stakeholders.

A facilitated process, *via* online virtual platforms, will bring together children and young people from different parts of the world in a creative collaboration to co-design and co-produce accessible communications ( Wake & Eames, 2013). Due to the multi-country and multi-continental reach of this study, three separate groups will be created, based on compatibility of time zones. Each group will develop ideas independently, while sharing and enhancing each other’s work in solidarity. The proposed geographical groupings are as follows: (a) Europe Group: Ireland and Scotland; (b) Asia Oceania Group: Taiwan, India, Australia, New Zealand-Aotearoa; and (c) Americas Group: Brazil, Canada, USA.

***Recruitment and selection of participants***. Promotion of and recruitment to the workshops will be advertised through social media, and further disseminated across establish globally connected networks of the project team and key stakeholders. This will include a flyer with information of the study details and how to get involved, using our study logo to promote our study (see Figure 4). To be eligible to participate in the workshops, members must be 10–17 years old and be able to participate fully using the working language of the relevant working group. Informed consent will be obtained from parents (or guardians) and assent from the child or young person. It is envisaged that there will be 10–15 members within each of the three workshop groups, based on the experience of the team of running such workshops, as this size of group is manageable, enables diversity of experiences and viewpoints, while enabling a sense of inclusion and active participation for all participants. CRAG members will also be given the option of becoming participants in these workshops.

***Co-design workshops in three geographical groups***. Following the Lundy model ( Lundy, 2007), these working groups will create new *spaces* where children can build informed understanding of the issues, build their confidence, build a sense of solidarity, understanding of the process, and preparedness for exercising their *voice* to an *audience* of professionals and policymakers.

The working group sessions will be facilitated by adults with suitable expertise in such process facilitation, who will be selected by project partners from their own teams or trusted associates. The adult facilitators will be required to have good understanding of children’s participation rights, intergenerational solidarity, skills for listening to, learning from, and collaborating with children, and awareness of the child safeguarding and ethical issues arising in such online collaborative working. In terms of the Lundy model, the adult role will be to support the child participants in developing and deploying a strong collective *voice.*

The outline structure, given in Table 3, is proposed for this process, which will be conducted virtually over a period of 2–3 months. However, guidance will be sought from the CRAG on the structure and organisation of the process, and facilitators will respond reflexively as the process develops, so some flexibility is anticipated. In terms of the Lundy model, the young participants are being supported in using their collective *voice* to put their ideas in front of the right *audiences*, to maximise their potential *influence* (or *impact*) on policy and practice. It is important to note that the adult partners and their organisations must hold themselves accountable to ensure follow-up on these action plans, undertaking what is feasible, and giving a reasoned account of what can and cannot be delivered.

**Table 3.** The overarching structure for the participatory workshops for work package 4.

*Workshop * *session*	Description and objective
1	Getting to know each other and establishing how we want to work together. Understanding and discussing the project aims, the goals of the working group process (see Figure 3), and proposed workplan.
2	Visual presentation of what has already been learnt from previous work packages (appropriately summarised). Discussion around key findings leading to brainstorming of initial ideas for new response initiatives.
3	Selection by consensus of at least one idea for a response initiative that can be developed into a deliverable package. First rough design of the proposed deliverable.
4	Each working group gets a visual presentation of the other two groups’ proposals for deliverables. They consider these proposals and develop feedback on how each idea can be further developed, enhanced, or made more effective.
5	Each working group receives the feedback on their initial proposal from the other two groups. Using this feedback, they further develop and refine their initial idea towards a final deliverable proposal. They prepare to pitch their idea to stakeholders in the next session.
6	“Pitch Day”: Stakeholders (policymakers, public officials and healthcare professionals) are invited to join the final session. Working group members pitch their proposal(s) to the panel and receive immediate feedback on the proposal, along with commitments on action from the panel. Examples include, to move the proposal forward to a development or implementation stage within their organisation or agency; or to engage with the working group to explore other options to chart a way forward.

*The structured programme of 6 sessions, after which each of the 3 groups will have developed a proposal for a new initiative, pitched this proposal and obtained feedback, including an action commitment, from the stakeholders.*

As this work package is qualitative in nature, the consolidated criteria for reporting qualitative research (COREQ) will be adhered to ensure rigour ( Tong *et al.*, 2007).

***Data analysis***. As outlined in Table 3, facilitation of this collaborative work involves the review of each working groups’ initial proposals, conducted amongst the wider working groups. Feedback received and incorporated learning from the process will facilitate the final proposal.

The co-design workshop process will generate two kinds of data:

- *Record of the co-production process:* This will be subjected to a qualitative thematic analysis to identify and explore aspects of the experience that can generate useful learning for refining this type of methodology in future projects.
- *Products or initiatives that will emerge as the outputs of the co-design process:* The working group members will develop an action plan to progress their initiatives but given the participatory nature of this work the nature of these initiatives and the steps to implementation have not been pre-defined.

No formal data analysis is envisaged. The process will, however, be critically evaluated by the participants and the researchers, and critical reflection on the methodological approach will be part of the broader research output.

**Expected outcome(s)**

There is a potential for a number of prototypes to be co-designed, co-developed and co-produced, but given the participatory nature if this work package, what these prototypes will be is not pre-defined, but flexible to the ideas and creative nature of the stakeholders ( Goodyear-Smith *et al.*, 2015). It is envisaged that at least 3 prototypes (one from each group) will be developed to a viable product. Findings will be summarised and presented to the CRAG and *via* the study website. Further, an open peer reviewed publication will be developed to disseminate the findings internationally.

### Dissemination of findings

A range of dissemination strategies will include sharing study findings with national and international research partners and networks.

The findings of the scoping review and rapid realist review will be disseminated through peer-reviewed open access publication. Additionally, findings will be presented at both national and international conferences and symposiums. For example, the team will facilitate a master class at the European Society for Traumatic Stress Studies (ESTSS) virtual conference on “Trauma and Mental Health during the Global Pandemic”. ESTSS is the umbrella organisation of European trauma societies ( https://estss.org/).

Other expected dissemination/engagement outputs will include plain English summaries, posted on the project’s dedicated website and over social media, to enable rapid wide dissemination of outputs.

Feedback to, and ongoing communication with, children and young people is also an important aspect of the dissemination plan. A variety of resources, including infographics and at least one short video or animations, will be developed and shared *via* the project website and our partners’ networks. As noted above in work package 4, the workshops will develop their own action plans to contribute to the dissemination of their outputs and maximise potential for policy impact. Through the wider project team, there is potential to consider translation of outputs into different languages according to their local contexts.

It is during this stage that the final element of the Lundy model comes into play: as the adult *audience* has engaged fully with the children’s *voice,* the dissemination of the resulting ideas are expected to have a real *influence* on policy and practice at different levels, such as through the dissemination of a policy brief.

### Study status

The CRAG is currently being setup and the two review work packages, WP1 and WP2, are ongoing. Data collection for WP3 is due to commence shortly in Autumn 2021.

## Conclusion

By recognising children as rights holders, active citizens, and agents of change, this project seeks to harness their creative expertise in order to develop rapid response deliverables that can be translated into policy and practice in response to the COVID-19 pandemic. It will thus generate practical interventions that can enhance the wellbeing of children during and after the pandemic and promote a range of positive community responses.

This is an ambitious goal, but this protocol sets out a route-map for how it can be achieved. It follows a logical structure, starting with the establishment of a sound knowledge base, then gathering a wide range of empirical data to capture and analyse children’s lived experience. The final stage is a collaborative co-design process, where adults and children work together on an equal basis to design and develop the deliverables. 

In the medium term, the project aims to provide a practical response to the current COVID-19 pandemic, through better understanding of how children’s actions can contribute to social mobilisation and help communities to adjust to changes. However, it will also generate a model of engagement with children as agents of change and builders of solidarity – locally and globally – that can be extended to future disasters or pandemics with significant long-term benefits.

## Data availability

### Underlying data

No underlying data are associated with this article.

### Extended data

Open Science Framework: COVISION. https://doi.org/10.17605/OSF.IO/UHQ4Y ( McAneney, 2021).

This project contains the following extended data:

- CRAG Assent Form – Child or young person (pdf). (Assent form for child/young person to be used for CRAG).
- CRAG Consent Form – Parent (pdf). (Consent form for parent to be used for CRAG).
- CRAG Information Sheet – Child or young person and parent (pdf). (Information sheet for child/young person and parent to be used for CRAG).
- WP3 Creative Data Assent Form – Child or young person (pdf). (Assent form for child/young person to be used online for WP3, creative data).
- WP3 Creative Data Consent Form – Parent (pdf). (Consent form for parent to be used online for WP3, creative data).
- WP3 Creative Data Information Sheet – Child or young person (pdf). (Information sheet for child/young person to be used online for WP3, creative data).
- WP3 Creative Data Information Sheet – Parent (pdf). (Information sheet for parent to be used online for WP3, creative data).
- WP3 Creative Data Online Survey (pdf). (Blank survey to be used online for WP3, creative data)
- WP4 Participatory Workshops Assent Form – Child or young person (pdf). (Assent form for child/young person to be used for participatory workshops).
- WP4 Participatory Workshops Consent Form – Parent (pdf). (Consent form for parent to be used for participatory workshops).
- WP4 Participatory Workshops Information Sheet – Child or young person and parent (pdf). (Information sheet for child/young person and parent to be used for participatory workshops).

Data are available under the terms of the Creative Commons Attribution 4.0 International license (CC-BY 4.0).

## Footnotes

^1^ In line with the United Nations Convention of the Rights of the Child (1989), the term children is being used to refer to people below the age of 18 years old.
