# Supplementary material for: Children as innovators: harnessing the creative expertise of children to address practical and psychosocial challenges of the coronavirus disease 2019 (COVID-19) pandemic – COVISION study protocol
Source: HRB Open Res. 2022 Feb 23;4:104. Originally published 2021 Sep 23. [Version 2] doi: 10.12688/hrbopenres.13290.2 (PMC8968158; doi:10.12688/hrbopenres.13290.2)
Supplement: Peer review history

# Peer review: Reviewer response for version 2

**DOI:** 10.21956/hrbopenres.14739.r31546

**Published:** 2022-03-30

**Authors:** Michelle Templeton

**Affiliations:** 1 Centre for Children's Rights, Queen’s University Belfast, Belfast, UK

This revised version of the study protocol clarifies the role of the Children’s Research Advisory Group (CRAG) within the study, including the time commitment and engagement level. The team provides a clearer description of their role within the work packages, analysis of the data and the co-design process. Some examples of potential outputs that could be achieved further underscore the support the CRAG will receive to develop their own ideas and interpretation, which may lead to the development of child friendly interventions. The team acknowledges the limitations of their choice of using digital means for recruitment, data collection and CRAG engagement and notes that these were necessitated due to COVID restrictions. It might be useful however to remain flexible and make more use of partners in other countries who may be able to engage with the CRAGs face-to-face as country specific restrictions ease.

Is the study design appropriate for the research question?

Yes

Is the rationale for, and objectives of, the study clearly described?

Yes

Are sufficient details of the methods provided to allow replication by others?

Partly

Are the datasets clearly presented in a useable and accessible format?

Not applicable

Reviewer Expertise:

Children’s Rights and participation; Participatory research methods and child-friendly co-production; Child protection and ethics; Relationships and Sexuality Education

I confirm that I have read this submission and believe that I have an appropriate level of expertise to confirm that it is of an acceptable scientific standard.

# Peer review: Reviewer response for version 1

**DOI:** 10.21956/hrbopenres.14466.r30652

**Published:** 2021-12-14

**Authors:** Michelle Templeton

**Affiliations:** 1 Centre for Children's Rights, Queen’s University Belfast, Belfast, UK

This study seeks to explore and understand children and young people’s coping strategies during Covid-19 to inform and contribute to societal change. Using the Lundy Model of child participation, the team aims to work with children and young people from around the world as advisors on the project to help them engage with young participants using ‘creative voice’ methodology. The study aims to increase the understanding of home and community-based strategies, particularly those addressing their sense of safety, calm, hope, self and community efficacy, and connectedness. Moreover, to identify ways in which children’s actions contribute to the capacity of others to adjust to the changes arising from the pandemic.

 This is an ambitious and exciting global project and established networks will be called upon to help, however, it is not clear who this will involve and how they will integrate and engage with the CRAGs, the data, and the dissemination plan. The virtual nature of the online CRAG meetings and the data collection, while practical for a global study, can also be restrictive to those who may find it difficult to express their creative voice, such as children with disabilities, children in care or detention, and refugee/migrant status children who may have low/no access to technology, how will the team ensure those children’s creative voices are sought?

 The CRAG methods section states that the CRAG will advise the researchers on the formulation of research questions, appropriateness of methods, design of data-gathering instruments, analysis and interpretation of findings, and/or design of dissemination materials and methods. While WP3 includes the CRAG developing child friendly material based on the findings, the ‘interpretation’ element of their role seems to have been lost. CRAG Methods section could be expanded to include information such as: 

- How will the team recruit and manage an international advisory group?
- How will the virtual meetings work, a mix of all ages (10-17), separate groups, etc.?
- Appropriate and accessible technology - How will they meet the needs of all the children in relation to access to equipment, time zones, etc.? Or is it assumed only children who already have access to equipment can join the CRAG? How will this impact on who is recruited and more importantly, who is not represented on the CRAG?
- Why are CRAG members invited to sit on the rapid review panel in WP1 but not the scoping review in WP2, particularly when they have already been identified as a ‘key stakeholder?’

Some of these considerations are answered in WP4 which has a more detailed rationale and methods section for the recruitment of a new set of children. I am confused as to why more children are needed for WP4. Should these not be the original CRAG members recruited at the outset? Essentially there are 2 separate CRAGS, one at the start of the project and one at the end, although original CRAG members will be invited to join the new group. It may be that some CRAG members drop out and the researchers may need to replenish the group when it gets to the end of the project. Also, how are the children in WP4 exercising their voice to an audience of professionals and policymakers? There is no indication as to how this will be done or set up, who are the ‘right’ audiences? It states that they will develop their own action plans but an example of the extent of their potential ‘influence’ or how the researchers will assist them in their activities, would be helpful.

 In WP3 (substantive research element with child participants), the project aims to end data collection when 150 pieces of data are received. Should a global project, qualitative in nature, seek to generate more data? Could a better ‘cut off’ point be determined such as using the responses from a number of countries, UN zones or the groupings identified in WP4? What will happen to the global element of the study if the majority of the 150 data units come from children in UK and Ireland or Europe? How can the team plan to ensure a more global reach?

 My reservations are:

- There is no explicit structure or processes set out for the CRAG recruitment and meetings, no timeline and no training activities, needs analysis, etc. How will the researchers build the capacities of the CRAG to engage at each stage with the issues at hand? For example, what training and other workshops do the CRAG members need before they can confidently sit on a literature review panel and contribute with other experts.
- The global nature of the reviews and engagement with children in CRAGs and as participants requires a team of researchers not only skilled in working, listening and advocating for children and young people, but experienced in co-production methodology that develops products with children, for children. Not to mention language translation and data management issues.
- The fact that the CRAG workshops will meet online excludes children who may lack access to, or be unable to, work technology. Offline methods working in collaboration with partner organisations could enhance the accessibility of the project to reach more marginalised children and get them involved as CRAG members or participants.

Is the study design appropriate for the research question?

Yes

Is the rationale for, and objectives of, the study clearly described?

Yes

Are sufficient details of the methods provided to allow replication by others?

Partly

Are the datasets clearly presented in a useable and accessible format?

Not applicable

Reviewer Expertise:

Children’s Rights and participation; Participatory research methods and child-friendly co-production; Child protection and ethics; Relationships and Sexuality Education

I confirm that I have read this submission and believe that I have an appropriate level of expertise to confirm that it is of an acceptable scientific standard, however I have significant reservations, as outlined above.

# Peer review: Reviewer response for version 1

**DOI:** 10.21956/hrbopenres.14466.r30620

**Published:** 2021-11-16

**Authors:** Siobhan O'Higgins

**Affiliations:** 1 Active* Consent Programme, School of Psychology, National University of Ireland, Galway, Ireland

The study has a very ambitious aim working with children across the world to better understand children and young peoples' coping with COVID 19 strategies and in so doing identify ways that children's actions can enhance the capacity of others to adapt to change. The planned process to achieve this is clearly laid out starting with the creation of a knowledge base by the researchers using 2 different methodologies of reviewing existing literature and evidence. The reviews will create a fuller understanding of protective mechanisms that mitigate the psychosocial risks children face in a pandemic. This knowledge base then creates a fuller understanding of all the psychosocial processes affecting children and young people in a time of uncertainty and unforeseen changes created by the pandemic; and what has worked to reduce those identified risks to children and finally how well such mechanisms may work. Armed with these global insights then the children and young people's voice is engaged based on Lundy's model of how to create the possibility for real participation in the process by those under 18.  

 The global nature of the reviews and engagement with young participants will require quite a team of participative researchers skilled in working and listening to children and young people. It is not clear who this will involve, what spaces will be used. The use of existing networks will undoubtedly help enormously with the process of Work package 3. Although I am not clear as to how the Children's Research Advisory Group - CRAG - will engage with the process of narrative analysis outlined in this work package. 

 The use of creative processes as a method to engage and then disseminate children's views on processes that support them through pandemics may exclude those not confident enough to be creative? Could other methods also be offered to engage their views and so ensure all members of the workshops in work package 3 are fully involved, Need more detail here on how the process will work of connecting the workshops across the 3 global areas.

 Work package 4 and throughout the whole process, working with their CRAG will hopefully ensure the real participation of children's voices in this process, but these only really appear from work packages 3 and 4. 

 My only real reservations are:

- In the use of social media to recruit and dissmentate - not being a 'digital native' the initial recruitment might then depend on parents noticing the recruitment strategies, rather than young people being initially interested themselves.
- By offering more detail on other methods for recruitment would reduce any criticism of class bias. 
- How will the process be managed if there is another lockdown in any of the 3 areas for workshop participants? I.e inclusion of a contingency plan to ensure that this project can be brought to fruition.
-I am left with no clear idea of timelines and time commitment - especially in terms of engagement by those on the CRAG and for the young people in the workshop. 

Other than that I believe this is a very exciting and worthwhile study and I look forward to reading how it all flows!

Is the study design appropriate for the research question?

Yes

Is the rationale for, and objectives of, the study clearly described?

Yes

Are sufficient details of the methods provided to allow replication by others?

Yes

Are the datasets clearly presented in a useable and accessible format?

Not applicable

Reviewer Expertise:

Children and young people , sexual health and wellbeing, sexual consent and participative research methods

I confirm that I have read this submission and believe that I have an appropriate level of expertise to confirm that it is of an acceptable scientific standard.
